# Application of a Low-cost, High-fidelity Proximal Phalangeal Dislocation Reduction Model for Clinician Training

**DOI:** 10.5811/westjem.59471

**Published:** 2023-08-25

**Authors:** Spencer Lord, Sean Geary, Garrett Lord

**Affiliations:** * Albany Medical Center, Department of Surgery and Department of Emergency Medicine, Albany, New York; † Massachusetts General Hospital, Department of Surgery, Boston, Massachusetts; ‡ Dattner Associates, New York, New York

## Abstract

**Introduction:**

Patients present to the emergency department (ED) relatively commonly with traumatic closed proximal interphalangeal joint (PIPJ) dislocations, an orthopedic emergency. There is a paucity of teaching models and training simulations for clinicians to learn either the closed dislocated dorsal or volar interphalangeal joint reduction technique. We implemented a teaching model to demonstrate the utility of a novel reduction model designed from three-dimensional (3D) printable components that are easy to connect and do not require further machining or resin models to complete.

**Methods:**

Students watched a two-minute video and a model demonstration by the authors. Learners including emergency medicine (EM) residents and physician assistant fellows assessed model fidelity, convenience, perceived competency, and observed competency.

**Results:**

Seventeen of 21 (81%) participants agreed the model mimicked dorsal and volar PIPJ dislocations. Nineteen of 21 (90%) agreed the model was easy to use, 21/21 (100%) agreed the dorsal PIPJ model and 20/21 (95%) agreed the volar PIPJ model improved their competency.

**Conclusion:**

Our 3D-printed, dorsal and volar dislocation reduction model is easy to use and affordable, and it improved perceived competency among EM learners at an academic ED.

Population Health Research Capsule What do we already know about this issue?

*Dorsal joint reductions are common orthopedic emergencies, yet there are few training models for clinicians.*

What was the research question?

*Does a novel low-cost, high-fidelity model dorsal joint reduction model improve competency?*

What was the major finding of the study?

*100% of participants agreed the dorsal proximal interphalangeal joint model (PIPJ) and 95% agreed the volar PIPJ model improved competency.*

How does this improve population health?

*An affordable education model enables medical trainees to learn the PIPJ dislocation reduction technique and, therefore, provide better patient care.*

## INTRODUCTION

Traumatic dislocations of the proximal interphalangeal joint (PIPJ) are among the most common types of finger dislocation. Dorsal dislocation is due to a lateral loading force with hyperextension leading to disruption of the volar plate, joint capsule, and composite ligaments leading to a dorsal dislocation.^1^^–^^3^ Volar dislocations, although much rarer, do occur and are often caused by compression and rotation with concomitant PIPJ flexion.^4^ Despite the prevalence of this injury within the trauma population, there are few teaching models available with no published practice assessment of the model trainers for clinicians.^5^^,^^6^

**Figure 1.**
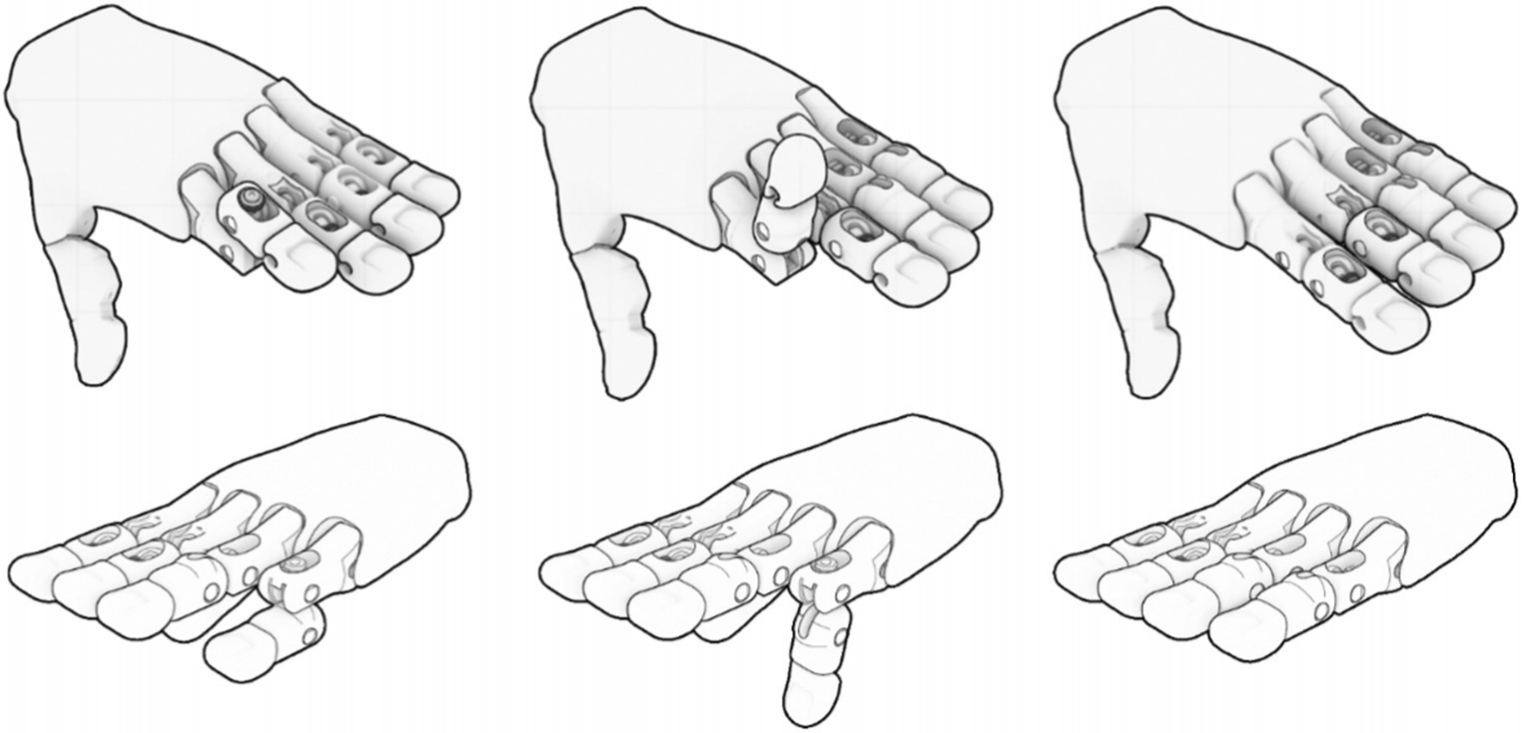
Proximal interphalangeal joint (PIPJ) complex dorsal dislocation with “exaggeration of deformity” method of the second finger (1A), hyperextension of the PIPJ (1B), and reduction back to the neutral position (1C). PIPJ volar dislocation of the fifth finger (1D), flexion reduction position of the PIPJ (1E), and reduction back to the neutral position (1F).

We provide a simple to print, assemble, and replace dorsal and volar reduction model created from three-dimensional (3D) printed components without requiring machining (Figure 1).^7^ Our model follows the principle of simple dorsal and volar dislocation that requires management using the exaggeration by deformity described by Roberts and Hedges et al in 1998.^8^ We developed a workshop to assess the model by evaluating model fidelity, model convenience, and clinician competency during a training module.

## METHODS

### Model Design

We developed a unique, interchangeable PIPJ model to practice reducing non-fractured PIPJ dorsal and volar dislocations. Specific details regarding model function and joint articulation are fully described within Lord 2022 et al.^7^ We developed a 3D-printed hand model using a palm base from the open-source Flexy-Hand prosthetic (e-NABLE global online community).^9^ We modified the second and third fingers to function as a dislocated PIPJ in closed complex dorsal dislocation (Figure 2.1–3) with reduction achieved by the exaggeration of deformity method, and the fourth and fifth fingers to function as a dislocated PIPJ in closed volar dislocation (Figure 2.4–6). The fingers are also detachable for reconfiguration and easy replacement (Figure 3).

**Figure 2.**
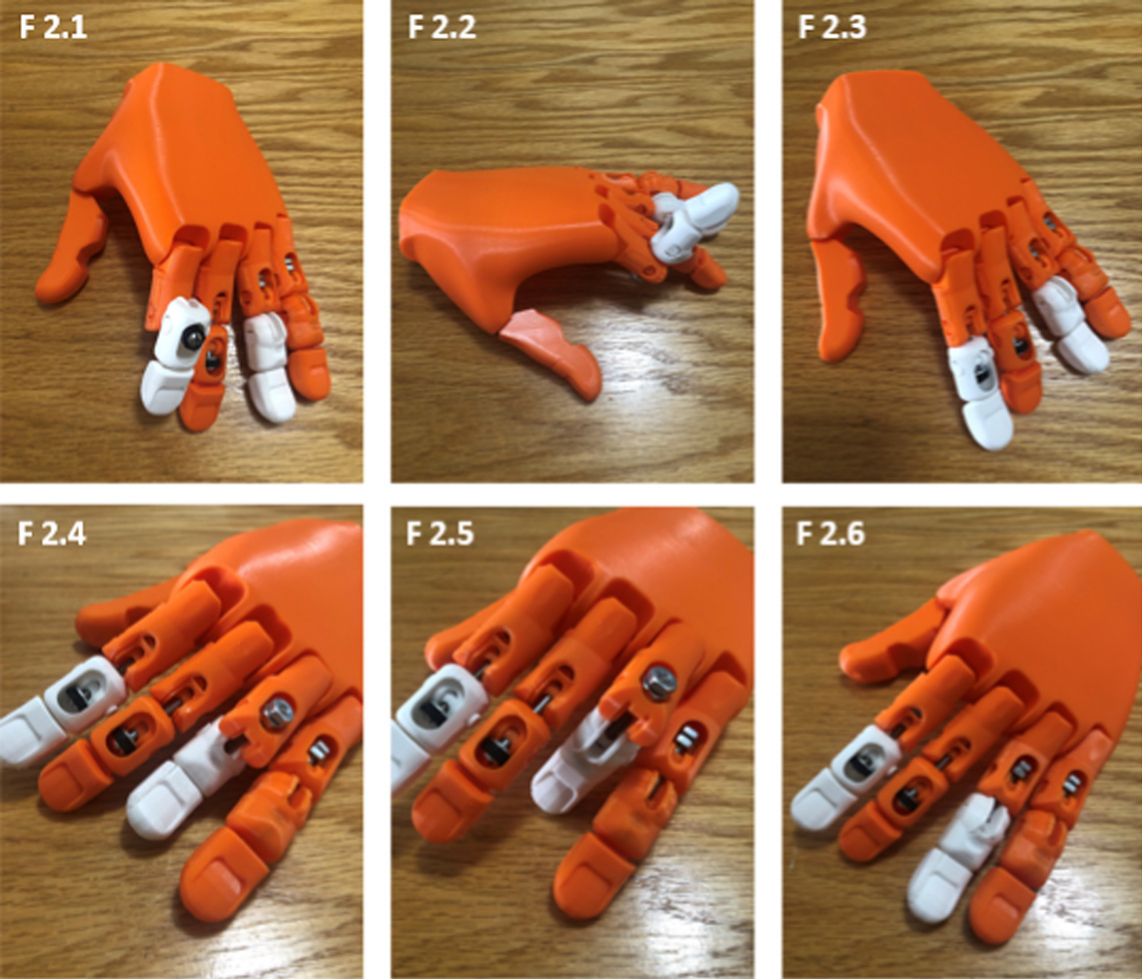
Proximal interphalangeal joint (PIPJ) complex dorsal dislocation with exaggeration of deformity method of the second finger (2.1), hyperextension of the PIPJ (2.2), and reduction back to the neutral position (2.3). PIPJ volar dislocation of the fourth finger (2.4), hyperflexion reduction position of the PIPJ (2.5), and reduction back to the neutral position (2.6).

**Figure 3.**
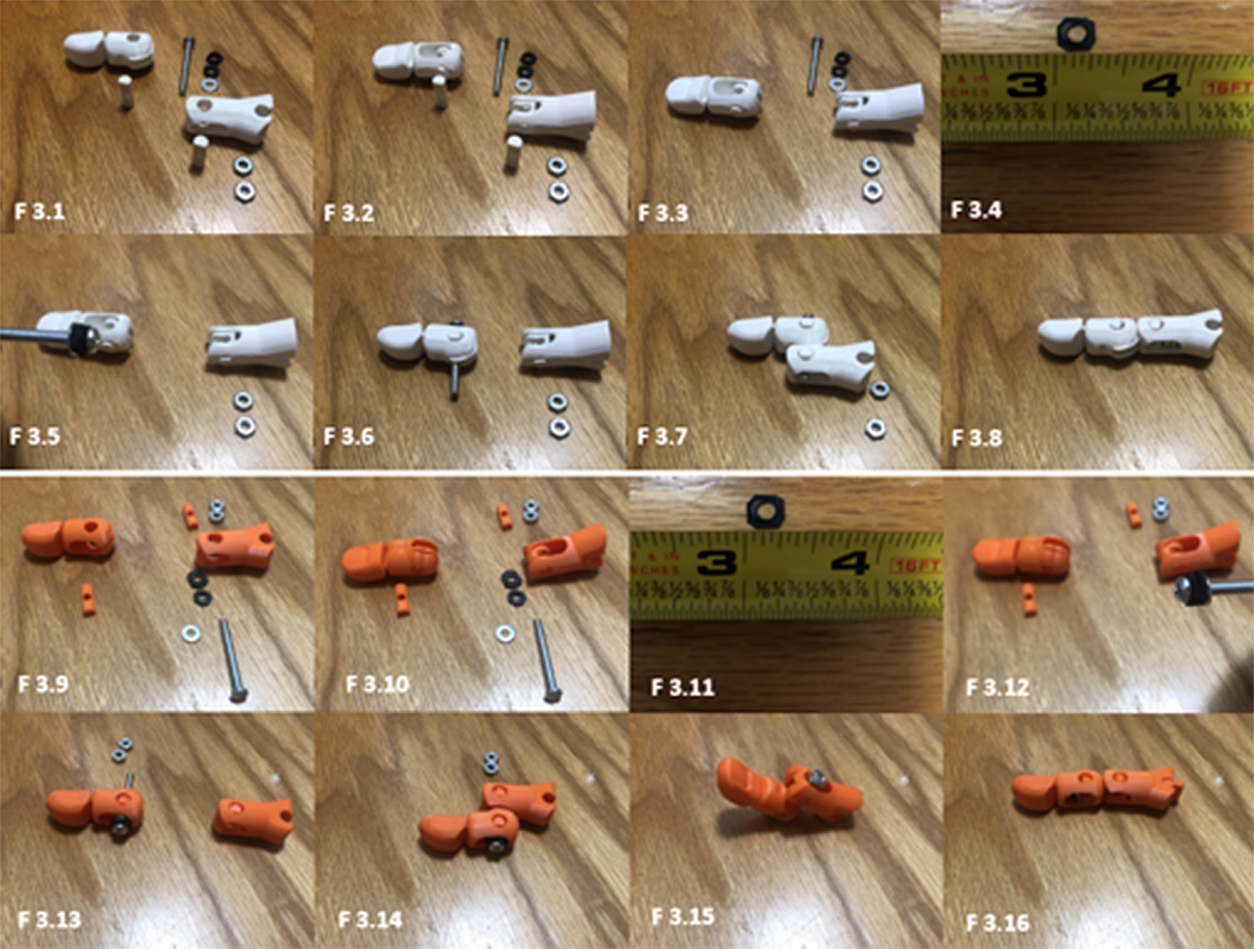
**3.1**. Shows all component parts of the dorsally dislocated finger (lateral view). **3.2**. Dorsal view. **3.3**. Place pin and align the central opening with openings in the middle and proximal finger portions. **3.4**. Modified neoprene washer cut ¼ in x ¼ in. **3.5**. Load two neoprene washers and a machine screw nut. **3.6.** Load the screw dorsally through the pin. **3.7**. pass screw through pin in proximal phalanges as shown and attach screw nuts in this position. **3.8**. Place in the reduced position to check resistance. **3.9**. Shows all component parts of the volar dislocated finger (lateral view). **3.10**. Dorsal view. **3.11**. Modified neoprene washer cut ¼ in x ¼. **3.12**. Load two neoprene washers and machine screw nut. **3.13**. Load the screw ventrally through the pin. **3.14**. Pass screw through pin in proximal phalanges as shown and **3.15**. attach screw nuts in this position. **3.16**. Place in the reduced position to check resistance.

**Figure 4.**
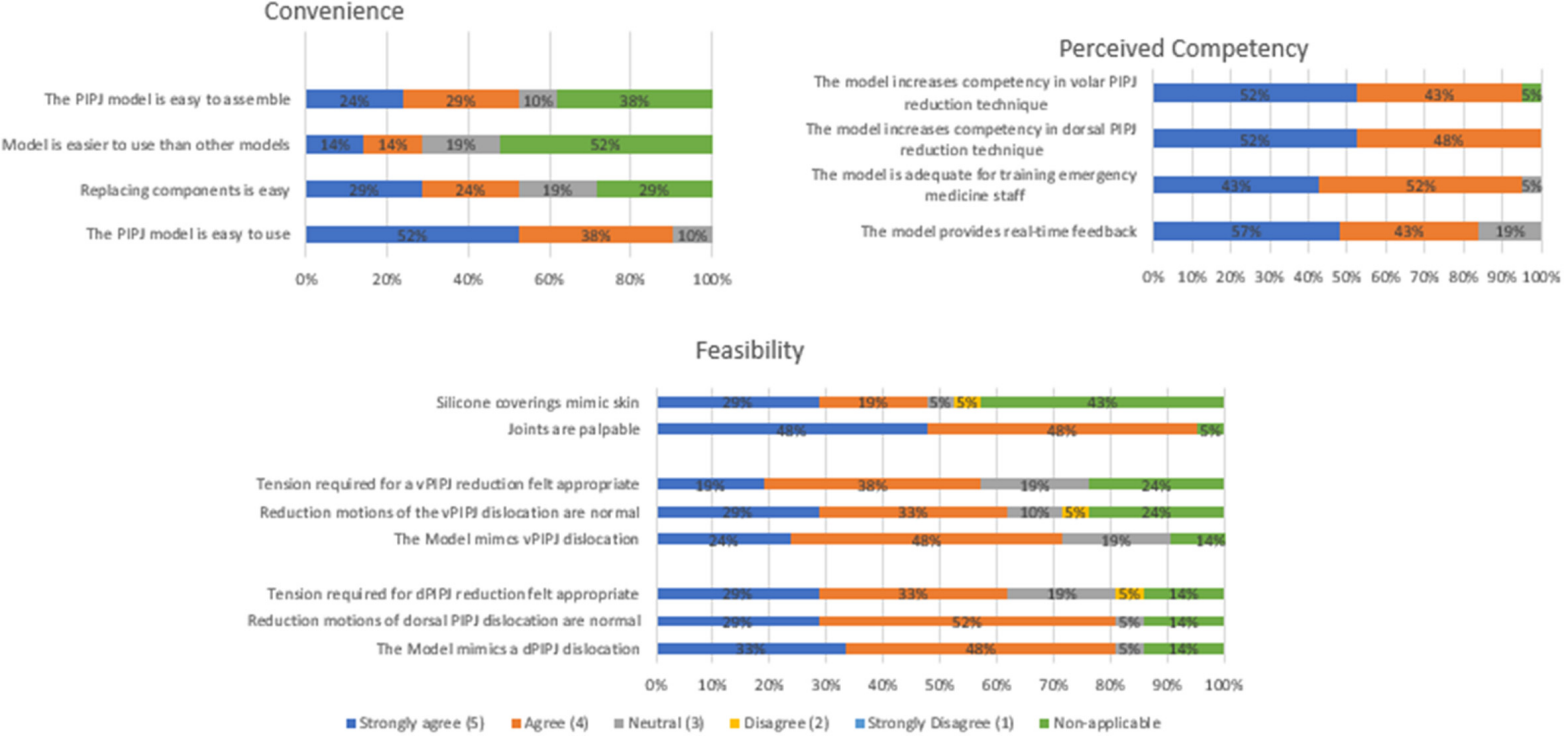
A graphical representation providing interpretation of model convenience, feasibility, and perceived training competency. *vPIPJ*, volar proximal interphalangeal joint; *dPIPJ*, dorsal proximal interphalangeal joint.

### Supplies

For model design we used Rhinoceros V6 software (McNeel and Associates, Seattle, WA), Ultimaker Cura version 4.9.0 G-CODE software (Ultimaker, Utrecht, Netherlands), a Creality Ender 3 3D printer (Creality, Schenzhen, China), and 1.75mm PLA filament (Hatchbox, Pomona, CA). Specifically for the joint apparatus we used a single #6–32 × 1–1/2in machine screw, two #6 flat washers, and four 5/32 neoprene washers from a hardware store, which we cut to size with a razor blade (Figure 4). The apparatus was hand tightened for added stability. Table 1 provides a breakdown of costs.

**Table 1.** Itemized list of model components including cost.

Item	Brand	Size	Unit cost ($)	Total cost
Machine screw	Everbilt	#6–32 × 1–1/2 in	0.42	4.98
Flat washer	Everbilt	#6	0.36	4.36
Machine screw nut	Everbilt	#6–32	0.11	1.28
Neoprene washer	Everbilt	5/32in	0.24	0.98
Razor blade	Stanley	n/a	1.97	1.97
1.75mm filament 1kg	Hatchbox	n/a	22.99	22.99
Rhinoceros V6 software	McNeel and Associates	n/a	195.00	195.00
Ultimaker Cura software	Ultimaker	n/a	Free	Free
G-CODE software	Ultimaker	n/a	Free	Free
Creality Ender 3 3D printer	Creality	n/a	179.99	179.99
Total Costs				411.55

#### Print Configuration

The entire print takes 33 hours and 25 minutes if each part is printed separately, with each finger taking 3 hours and 28 minutes (proximal, middle, distal, pin, and hinge). An entire hand requires the production of four proximal, four middle, and four distal finger components, along with eight pins and four hinge connectors along with a single palm and single thumb. The properties of all printed components are included in Table 2. The.stl print is available, open source, at https://grabcad.com/library/proximal-phalanx-traction-model-1. We provide a step-by-step assembly of the dorsal dislocation and volar dislocation. Supplemental file 4 includes how to use Cura Ultimaker software to upload your.stl file and assign your printer.

**Table 2.** Properties of all printed components.

Item	Print time	Filament weight (g)	Filament length (m)	Wall thickness (mm)	Top/bottom thickness (mm)	Infill%/type	Layer height (mm)
Palm	18 hours 42 min	(134g)	45m	0.8mm	1.0mm	50%/Cubic	0.2mm
Proximal	1 hour 23 min	8g	2.81m	0.8mm	1.0mm	50%/Cubic	0.2mm
Middle	1 hour 5 min	6g	2.01m	0.8mm	1.0mm	50%/Cubic	0.2mm
Distal	33 min	4g	1.36m	0.8mm	1.0mm	50%/Cubic	0.2mm
Pin	6 min	<1g	0.13m	0.8mm	1.0mm	50%/Cubic	0.2mm
Hinge	15 min	1g	0.27m	0.8mm	1.0mm	50%/Cubic	0.2mm
Thumb	1 hour 51 min	11g	3.74m	0.8mm	1.0mm	50%/Cubic	0.2mm

*g*, gram; *m*, meter; *mm*, millimeter.

### Model Assembly

The following figures show the stepwise assembly of both the dorsal dislocation and volar dislocation (Figure 3). A step-by-step video assembly of the dorsal PIPJ reduction is available at (https://youtu.be/TkEyc3R2p9s) and volar PIPJ reduction (https://youtu.be/_MCwHHbP-Sk). An exploded assembly model is described in Lord et al 2022.^7^

### Study Design

This was a prospective observational study performed at a single, Level I trauma center emergency medicine (EM) residency program during the months of November 2021 and March 2022. All study participants were current EM residents and physician assistants, who were given a live demonstration of how to perform a volar and dorsal reduction by the exaggeration method on the provided teaching model [https://www.youtube.com/watch?v=i1XPiA3GYmQ]. Participants were assessed for observed competency using a stepwise assessment, and they in turn assessed the model’s fidelity and convenience, as well as their perceived competency. (See supplemental 1, supplemental 2, and supplemental 3.)^10^^,^^11^ Participants were not assessed for previous competency. Institutional review board approval was granted for an anonymous survey of an educational experience with dissemination of survey results for publication.

### Workshop

Following the demonstration, participants were assessed for observed competency. The total teaching section took place during a 90-minute scheduled period within residents’ protected educational time. Participants were given two attempts to practice both the volar and dorsal dislocation model before assessment attempt. Procedural steps were scored based on whether the task was completed or there was hesitancy or omission/failure. We determined that hesitancy would be defined as a pause >3 seconds. Omission was defined as skipping a step, and we defined failure as inappropriately deforming or breaking the model. Following the workshop, participants were asked to complete a short survey using a six-point Likert scale (5 strongly agree, 4 agree, 3 neutral, 2 disagree, 1 strongly disagree, non-applicable) pertaining to questions regarding model fidelity, convenience, and competency.^11^ The workshop director was available for questions after the participants attempted the model and completed the questionnaire and was available to rebuild the model if participants could not do so themselves.

## RESULTS

During the study period a total of 21 participants comprising 19 residents (six postgraduate year [PGY] 1, seven PGY 2, and six PGY 3) and two physician assistant fellows completed both the dorsal and volar PIPJ reduction model. All participants consented to allow use of their data for research purposes. Data analysis was performed with Excel 2006 (Microsoft Corp, Redmond, WA). Figure 4 provides a graphical representation of model feasibility, convenience, and perceived competency.

### Model Feasibility

All 21 participants were included in the feasibility portion. Nineteen of 21 (95%) agreed the joints were palpable on exam; 2/21 (5%) did not answer the question. Seventeen of 21 (81%) participants either agreed or strongly agreed that the model mimicked a dorsal proximal interphalangeal joint (dPIPJ) dislocation, and 17/21 (81%) agreed or strongly agreed it mimicked a volar proximal interphalangeal joint (vPIPJ) dislocation. Also, 13/21 (62%) and 12/21 (57%) agreed or strongly agreed that tension required for successful joint reduction of the dPIPJ and vPIPJ was realistic. Seventeen of 21 (81%) participants agreed the model mimicked a dorsal and volar PIPJ dislocation. Nineteen of 21 (90%) agreed the model was easy to use, and 100% agreed the model improved their competency.

### Convenience

All 21/21 participants were included in the convenience portion. Nineteen (90%) agreed or strongly agreed that the model was easy to use. Of the participants who had practiced on a finger reduction model before, 6/10 (60%) agreed or strongly agreed this model was easier to use, 4/21 (19%) thought the model was neutral or no different in use compared to other models, and 11/21 (52%) selected “non-applicable” as they had no model to compare. Six of the 21 participants (29%) did not have to replace components during their testing and, thus, they responded “non-applicable” to the question.

### Perceived Competency

All 21/21 participants were included in the perceived competency portion. Twenty-one (100%) agreed the dPIPJ model improved their competency in dPIPJ reduction technique. Nineteen (95%) agreed the vPIPJ model improved their competency in vPIPJ reduction technique. Nineteen (95%) agreed the model is adequate for training EM staff.

### Observed Competency

**Figure 5.**
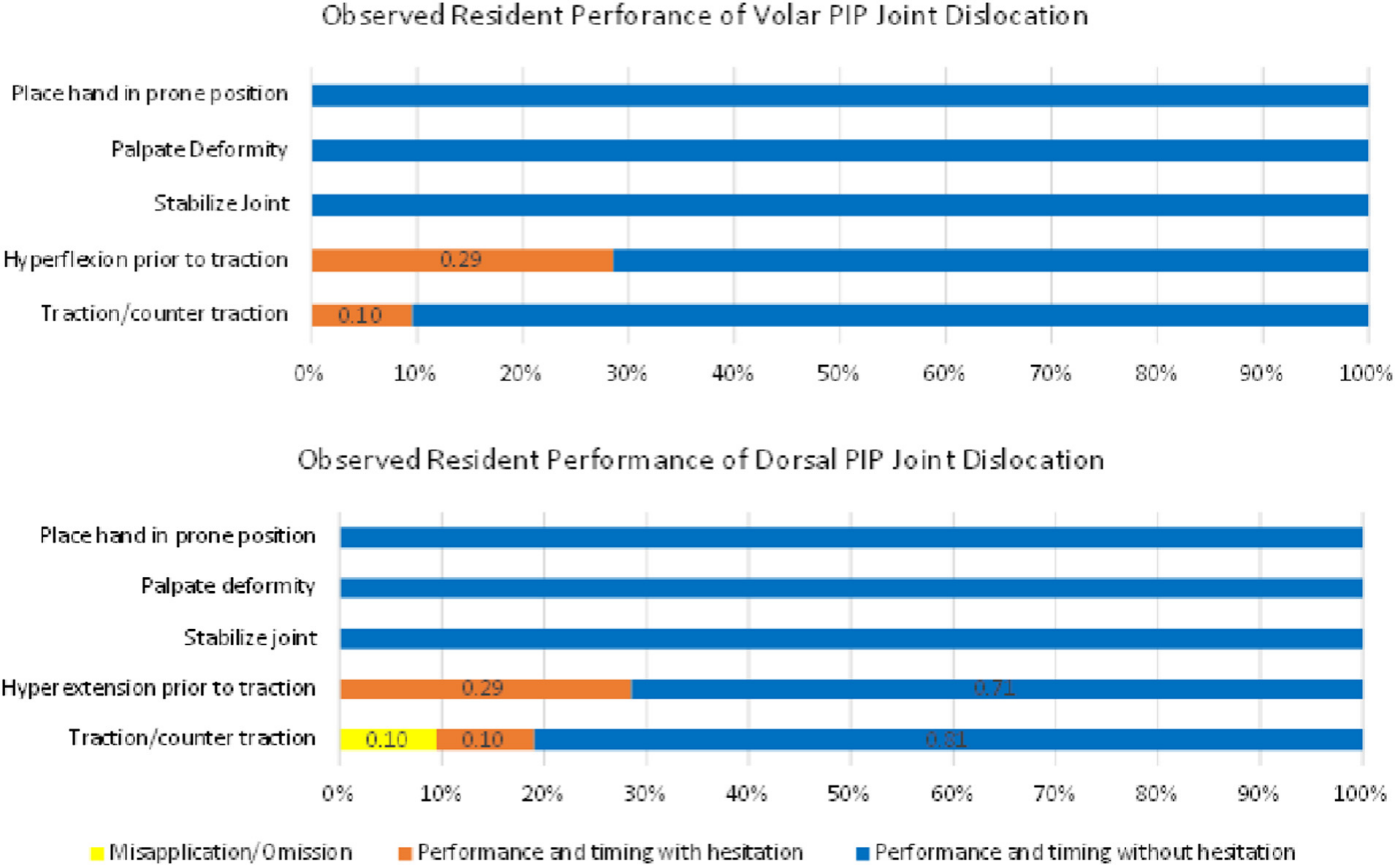
Observed competency of the volar proximal interphalangeal joint (PIPJ) reduction model and dorsal PIPJ reduction model.

All 21 participants were included in the observed competency portion (Figure 5). In both the volar and dorsal reduction groups, all participants placed the hand in a prone position, palpated the deformity and stabilized the joint prior to reduction using the correct practice without hesitation. Fifteen of 21 (71%) performed correct hyperextension and hyperflexion without hesitation, and 6/21 (29%) performed hyperextension and hyperflexion but with hesitancy. In the dPIPJ dislocation, 17/21 (81%) performed traction/countertraction correctly and without hesitation, 2/21 (10%) performed traction/counter traction correctly but with hesitancy, and 2/21 (10%) performed traction/countertraction incorrectly resulting in detachment of the metacarpal-phalangeal joint from the hand model. In the vPIPJ dislocation, 19/21 (90%) performed traction/countertraction correctly without hesitancy. Two of 21 (10%) performed traction/countertraction correctly but with hesitation.

## DISCUSSION

This is the first low-cost, high-fidelity PIPJ reduction model published in the medical literature describing a reproducible fidelity study. The model has removable and replaceable components without the requirement of resin mold production or machining. This model also provides appropriate competency training in a low-stress, teaching environment for learners. The model provides convenience regarding assembly, replacement, and use. The model mimics real-life dPIPJ and vPIPJ simple dislocations and shows both perceived and observed competency with practice. With few misapplications/omissions noted during the observed performance, our model avoids the “fidelity trap” (assuming participants “learn in proportion to the level of realism”) that can occur with high-fidelity teaching models.^12^^,^^13^

Our design not only depicts a dislocation, but it also requires anatomically correct motions to successfully reduce the joint into the correct position. The unique articulation surfaces prevent “cheating,” enable learners to experience the significant tension and pressure needed to successfully reduce the joint and allow for an easy reset to the dislocated position for the next participant. Our model provides improvement over currently available products due to tested repeatability, relative affordability, and a unique joint apparatus to prevent incorrect technique. It is the first model of its kind made from reusable, replaceable 3D-printed components from a personal 3D printer and easily available components from a hardware store.

Future studies will focus on addressing limitations, specifically creating a more realistic finger cover to better replicate the procedure or trialing different silicone thicknesses without adding significantly more construction requirements for the trainee. While the silicone covering we chose was an affordable option, it often caught itself in the joint making the reduction attempt impossible. Other anticipated model innovations include testing and refining appropriate force requirements during reduction, ideally using a quantitative force transducer. Further studies are needed to assess different clinician groups who perform this procedure including prehospital medical services, military personnel, more senior emergency clinicians, and non-emergency clinicians including orthopedic and plastic surgeons.

Three-dimensional anatomical learning has been shown to be a more effective studying tool compared to 2D technologies.^14^ Further, 3D teaching models are noninferior to cadaveric teaching, are often cheaper, and potentially more accessible.^15^^–^^17^ Simple and even complex joint reductions can be performed out of hospital, and studies have shown the relative ease and safety of prehospital joint reductions.^18^^–^^20^ Procedural training using high-fidelity teaching models provides trainees with non-inferior procedural training in the absence of expensive mannequins. A recent meta-analysis found that operative time and complication rates improved when using 3D models for surgical planning, and a large multispecialty review highlighting the role of 3D printing in complex medical procedural planning and training showed improved competency.^21^^,^^22^ There is a growing field in preoperative planning in orthopedic oncology, and orthopedic trauma surgery.^23^^–^^25^

## LIMITATIONS

The model does have limitations. Namely, there are no models available for purchase nor are there published training models to compare.^5^^,^^6^ Therefore, while participants deemed the model easy to use, we could not objectively compare feasibility, competency, and convenience to other models. The model also takes time to assemble due to the intentional metacarpophalangeal joint dislocation mechanism and assembly by non-machined parts. While most participants believed the model represented a realistic PIPJ dislocation, to maintain stability we reduced the degree of rotational displacement of the dislocated joint, therefore reducing model fidelity. Furthermore, our dorsal dislocation model is more applicable when there is a bayonet deformity (ie, when the condyle of the proximal phalanx is no longer in contact with the base of the middle phalanx). In the absence of bayonetting, the degree of hyperextension that the model requires is not always needed in the clinical setting.

While the joint apparatus was functional it was difficult to use and articulate with the purchased silicone coverings; half the participants asked to remove the silicone finger cover prior to participating in the evaluation; thus, learners were unable to practice in the environment of a truly closed dislocation. While we attempted to assess observed competency, we did not compare outcomes between those who had reported performing a dorsal dislocation reduction (7/21) (33%) or volar dislocation reduction (2/21) (10%). Due to the relatively small sample size of this study, we did not ask about total number of attempted live reductions or success rate of attempted live procedures. Further, with the small sample size and with most participants being residents, this study lacks generalizability.

While printing of the model was replicable, a general knowledge of Cura Ultimaker software is needed to complete the print, which for the non-engineers among the authors, took two hours of online tutorial instruction to understand and be self-sufficient. This may not be an accurate length of time required to learn the software as we did not assess this in our study.

Of the participants who did not agree or strongly agree that the traction was realistic, 40% attributed this to believing the tension was too tight. We did not confirm whether this was the reason two participants did not agree the model was easy to use, nor whether the reason four participants did not agree or strongly agree the model mimicked vPIPJ and dPIPJ dislocations.

We did not use a quantitative force transducer as we could not find a simple way to incorporate it into the study model. It would be extremely beneficial to further refine the device to achieve more accurate representation for forces required for reduction. Also, it should be noted that 8/21 (38%) documented “non-applicable” for the convenience question—“The PIPJ model is easy to assemble”—even though each participant had to attach the fingers to the hand model prior to use.

## CONCLUSION

Our unique proximal interphalangeal joint dislocation model provides a reproducible, easy-to-print product that can be used within the learning environment to train practitioners. The model is easy to assemble and provides a joint articulation to train learners for both dorsal and volar PIPJ dislocations.

## Supplementary Information








