# Supplementary material for: Application of a Low-cost, High-fidelity Proximal Phalangeal Dislocation Reduction Model for Clinician Training
Source: West J Emerg Med. 2023 Aug 25;24(5):839–46. doi: 10.5811/westjem.59471 (PMC10527832; doi:10.5811/westjem.59471)
Supplement: Supplementary file 1 [file wjem-24-839-s001.docx]

Supplemental 1 Form A:

Number #: ________

Consent:

______ By marking this line you CONSENT to your responses being used for research purposes

_______ By marking this line you DO NOT CONSENT to your responses being used for research purposes

Print name: ______________________________ Signature: ________________________

Joint Reduction of a dorsally dislocated PIPJ

1. Equipment preparation.
2. Place hand in prone position.
3. Clean injection site.
4. Perform a digital nerve block.
5. Palpate the deformity including the proximal portion of the middle phalanx.
6. Stabilize the proximal phalanx with your nondominant hand and stabilize the middle phalanx with your dominant hand.
7. Initial movement is hyperextension at the PIP joint.
8. Traction of the middle phalanx towards the neutral position (with flexion motion) and counter traction on the proximal phalanx.
9. Stabilize Joint in the neutral position with splint.

Joint Reduction of the volar dislocated PIPJ

1. Equipment preparation.
2. Place hand in prone position.
3. Clean injection site.
4. Perform a digital nerve block.
5. Palpate the deformity including the proximal portion of the middle phalanx.
6. Stabilize the proximal phalanx with your nondominant hand and stabilize the middle phalanx with your dominant hand.
7. Initial movement is flexion at the PIP joint.
8. Traction of the middle phalanx towards the neutral position (with extension motion) and counter traction on the proximal phalanx.
9. Stabilize joint in the neutral position with splint.
