# Supplementary material for: Application of a Low-cost, High-fidelity Proximal Phalangeal Dislocation Reduction Model for Clinician Training
Source: West J Emerg Med. 2023 Aug 25;24(5):839–46. doi: 10.5811/westjem.59471 (PMC10527832; doi:10.5811/westjem.59471)
Supplement: Supplementary file 2 [file wjem-24-839-s002.docx]

Form B

Procedural step skills #____________

Required procedure Dorsal PIP Joint dislocation

| Equipment preparation | Misapplication/Omission %(n) | Correct performance and timing with hesitation %(n) | Correct performance and timing without hesitation %(n) |
| --- | --- | --- | --- |
| Place hand in prone position |  |  |  |
| Clean injection site |  |  |  |
| Perform a digital nerve block |  |  |  |
| Palpate deformity |  |  |  |
| Stabilize Joint |  |  |  |
| Initial Movement is Hyperextension |  |  |  |
| Traction and Counter traction |  |  |  |
| Stabilize joint with splint |  |  |  |

| Equipment preparation | Misapplication/Omission %(n) | Correct performance and timing with hesitation %(n) | Correct performance and timing without hesitation %(n) |
| --- | --- | --- | --- |
| Place hand in prone position |  |  |  |
| Clean injection site |  |  |  |
| Perform a digital nerve block |  |  |  |
| Palpate deformity |  |  |  |
| Stabilize Joint |  |  |  |
| Initial Movement is Flexion |  |  |  |
| Traction and Counter traction |  |  |  |
| Stabilize joint with splint |  |  |  |

To match patients between their skills assessment and qualitative analysis. Everyone who participates will be assigned a number at random which will be used to match between the skills assessment and qualitative analysis. The skills assessment will be performed on paper. This paper will be in a locked box located in P333 under the supervision of Sean Geary. The qualitative analysis will be performed on Qualtrics and the randomized number given earlier will be used to match the participant to their skills assessment. The quantitative analysis will be based on a survey provided by Lord et al 2021 and Sullivan et al. 2018.
