# Supplementary material for: Application of a Low-cost, High-fidelity Proximal Phalangeal Dislocation Reduction Model for Clinician Training
Source: West J Emerg Med. 2023 Aug 25;24(5):839–46. doi: 10.5811/westjem.59471 (PMC10527832; doi:10.5811/westjem.59471)
Supplement: Supplementary file 3 [file wjem-24-839-s003.docx]

Supplemental 3: Form C

Survey

Q1. By clicking the following you consent to the use of your anonymous survey data for research purposes

Q 2. Please provide your assigned number___ (you will not be able to advance without providing your number

Q 3. Level of training (single choice):

- PGY 1
- PGY 2
- PGY 3
- Junior EM faculty (practicing less than five years after residency)
- Senior EM faculty (practicing more than five years after residency)
- Nurse
- Nurse Practitioner
- Physician Assistant
- Physician Assistant Fellow

Q4. Have you performed a Dorsal PIPJ reduction before

- Yes
- No

Q5. Have you performed a volar PIPJ reduction before

- Yes
- No

User survey

| Question | Strongly agree (5) | Agree (4) | Neutral (3) | Disagree (2) | Strongly Disagree (1) | N/A |
| --- | --- | --- | --- | --- | --- | --- |
| Fidelity | | | | | | |
| The model mimics a dorsal PIPJ dislocation |  |  |  |  |  |  |
| Joints are palpable |  |  |  |  |  |  |
| Reduction motions of the dorsal PIPJ dislocation are normal |  |  |  |  |  |  |
| Tension required to reduce the dorsal PIPJ felt appropriate |  |  |  |  |  |  |
| The silicon coverings mimic skin |  |  |  |  |  |  |
| The model mimics a volar PIPJ dislocation |  |  |  |  |  |  |
| Joints are palpable |  |  |  |  |  |  |
| Reduction motions of the volar PIPJ dislocation are normal |  |  |  |  |  |  |
| Tension required to reduce the volar PIPJ felt appropriate |  |  |  |  |  |  |

Competency

| Question | Strongly Agree (5) | Agree (4) | Neutral (3) | Disagree (2) | Strongly Disagree (1) | N/A |
| --- | --- | --- | --- | --- | --- | --- |
| The dorsal PIPJ joint model with proper reduction motions |  |  |  |  |  |  |
| The volar PIPJ joint model with proper reduction motions |  |  |  |  |  |  |
| The model provider real-time feedback |  |  |  |  |  |  |
| This model is adequate for training EM staff |  |  |  |  |  |  |
| This model increases competency in PIPJ reduction techniques |  |  |  |  |  |  |

Convenience

| Question | Strongly Agree (5) | Agree (4) | Neutral (3) | Disagree (2) | Strongly disagree (1) | N/a |
| --- | --- | --- | --- | --- | --- | --- |
| The PIPJ model is easy to use |  |  |  |  |  |  |
| Replacing components is easy |  |  |  |  |  |  |
| The model is easier to use than other models |  |  |  |  |  |  |
| The PIPJ model is easy to assemble |  |  |  |  |  |  |

Qualtrics link:

<https://amc.az1.qualtrics.com/jfe/form/SV_3VLrmYri6IBqf1c>
