# Supplementary material for: Application of a Low-cost, High-fidelity Proximal Phalangeal Dislocation Reduction Model for Clinician Training
Source: West J Emerg Med. 2023 Aug 25;24(5):839–46. doi: 10.5811/westjem.59471 (PMC10527832; doi:10.5811/westjem.59471)

**Generating the 3D Printer Settings**

The 3D print software used in this article is Ultimaker Cura. A free download is available at: <https://ultimaker.com/software/ultimaker-cura>

**Selecting the 3D Printer**

1. Upon opening Cura, navigate to **Settings > Printer > Add Printer**


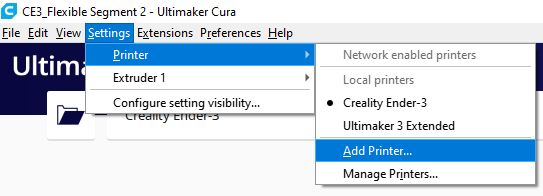


1. If working with a non-connected printer, select the appropriate 3D printer from the drop-down menu, and click **Add.**


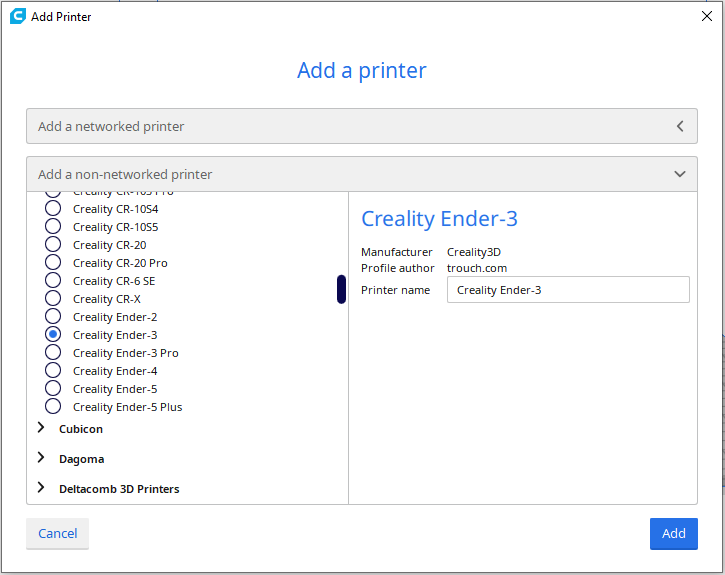


**Setting the Primary Extruder, Nozzle Size, and Print Material**

1. If working with a multiheaded extruder, go to **Settings > Extruder 1 > Set as Active Extruder**


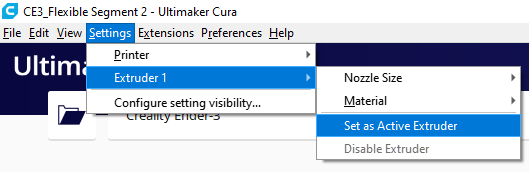


1. Next, go to **Settings > Extruder 1 > Nozzle Size**, and select the appropriate size nozzle.


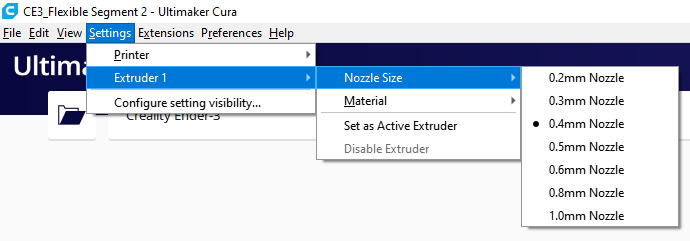


1. If the nozzle size is unknown, this can often be found engraved on the nozzle.

(The print files associated with this model are optimized for a 0.4mm nozzle.)

1. To select the material being use go to **Settings > Extruder > Material > Generic** and select the filament type being used. If the drop-down list under Materials contains the brand and filament type, select the appropriate one.


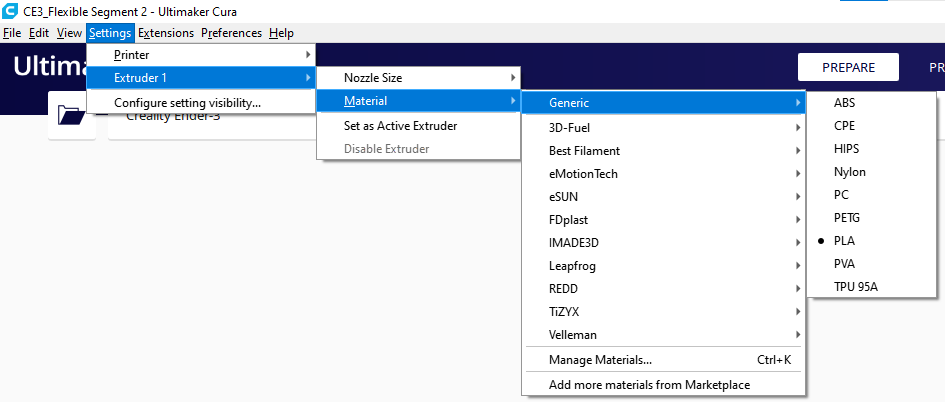


**Adjust the Print Settings**

1. Along the bar at the top of the workspace, click on the **Quality Settings** and a **Print Settings** dialog will appear. In the **Profile** field, select the desired Quality. (The print files have been optimized for Standard Quality)


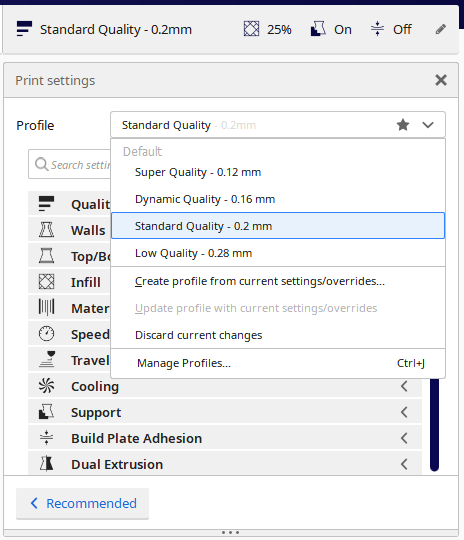


**Preparing the File to Print**

With the Settings Adjusted the STL model file can be imported and converted into a G-Code file to send to the 3D printer.

**Import the Geometry**

1. Navigate to **File > Open** and proceed to open the associated STL file.


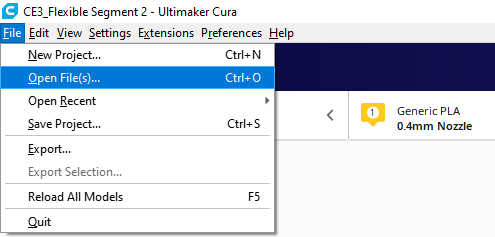


1. The selected file will show as a preview in a simulated Print Bed. Use the trackpad to zoom in and out, and use the arrow keys to rotate your view.


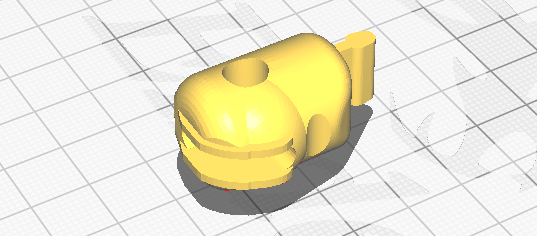


**Generate the Print File**

1. With the STL file loaded, select the **Slice** button on the lower right portion of the window.


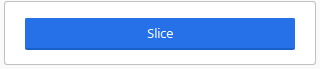


1. To review the g-code path, select the **Preview** button at the top of the viewing window. This view details the tool-path and differentiates the support material, outer layer, surfaces, and inner structure. If there is an error with the print file, it can be previewed here.


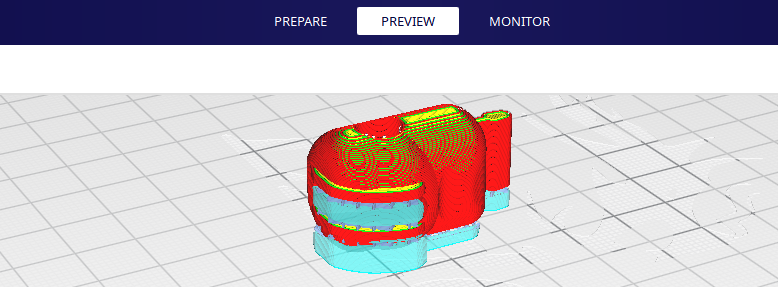


1. If the preview shows no errors, select the **Save to Disk** button on the lower right portion of the window. The file is now ready to be send to the 3D printer. The **Save to Disk** dialog will give an approximate run-time and amount of material used.


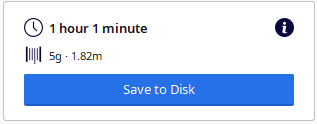

Supplement: Supplementary file 4 [file wjem-24-839-s004.docx]
